# Sensory processing and neurodevelopmental disorder traits in 4- to 5-year-old Dutch children adjusted for sex, neurodevelopment in infancy and IQ

**DOI:** 10.1007/s00787-026-03110-7

**Published:** 2026-07-06

**Authors:** Selena J. Rosinda, Pieter J. Hoekstra, Mijna Hadders-Algra, Kirsten R. Heineman, Annelies de Bildt

**Affiliations:** 1 University of Groningen, University Medical Center Groningen, Department of Paediatrics, Beatrix Children’s Hospital, Division of Developmental Neurology, Groningen, the Netherlands; 2 Accare Child Study Center, Groningen, the Netherlands; 3 University of Groningen, University Medical Center Groningen, Department of Psychiatry, Groningen, the Netherlands; 4 The Research School of Behavioural and Cognitive Neurosciences, University of Groningen, Groningen, the Netherlands; 5 University of Groningen, University Medical Center Groningen, Department of Paediatric Neurology, Groningen, the Netherlands; 6 Health in Context Research Institute, University Medical Center Groningen, University of Groningen, Groningen, the Netherlands

**Keywords:** Sensory processing, Autism Spectrum Disorder, Attention Deficit Hyperactivity Disorder, Developmental Coordination Disorder, Dimensional approach

## Abstract

****Purpose**:**

Sensory processing is an important aspect of children’s development and is closely linked to other developmental domains. We investigated sensory processing and its relation to the severity of neurodevelopmental disorder traits (i.e., traits associated with Autism Spectrum Disorder, Attention Deficit Hyperactivity Disorder, Developmental Coordination Disorder) in a sample of 4-5-year-old children from the general population, with an oversampling of children with atypical infant neurodevelopment.

****Methods**:**

The sample contained 557 Dutch 4-5-year-old children. Sensory processing was assessed with the Sensory Profile. Neurodevelopmental disorder traits were measured with parent- and teacher-reported questionnaires (Social Responsiveness Scale, Strengths and Difficulties Questionnaire, Conners Parent Rating Scale, Conners Teacher Rating Scale) and an assessment of motor abilities (Movement Assessment Battery for Children). Multivariate polynomial regression analyses were conducted to examine the relation between sensory processing and the severity of neurodevelopmental disorder traits. The child’s sex, neurodevelopment in infancy (as assessed with the Standardized Infant Neuro Developmental Assessment and Infant Motor Profile) and intelligence level at 4–5 years (as measured with the Wechsler Primary and Preschool Scale of Intelligence) were included as covariates.

****Results**:**

In 35–44% of the children sensory processing difficulties (varying per sensory processing quadrant) were present. Missing, seeking, avoiding and/or being sensitive to sensory stimuli was associated with more social responsiveness problems, hyperactive and/or inattentive behaviour and motor difficulties.

****Conclusion**:**

Sensory processing difficulties are prevalent in a sample of 4- to 5-year-old children, enriched for the presence of atypical infant neurodevelopment. Additionally, specific types of sensory processing difficulties are related to more severe neurodevelopmental disorder traits.

**Supplementary Information:**

The online version contains supplementary material available at https://doi.org/10.1007/s00787-026-03110-7.

## Introduction

Sensory processing is an important aspect of children’s development as afferent information provides the opportunity to learn [1]. Specifically, the 4E (embodied, embedded, enacted, extended) cognition framework stresses that cognition develops based on interactions between the brain, body and active engagement with the environment [2]. In these interactions, sensory processing plays an essential role. Additionally, the cerebellar connectome hypothesis proposes that the cerebellum, which integrates sensory stimuli, is related to cortical and subcortical networks. This indicates that sensory processing plays an important role in motor, cognitive and emotional development [3, 4]. Due to the suggested impact of sensory processing difficulties on multiple developmental domains and participation in daily life activities [5] it is important to examine sensory processing in children.

Two dimensions are involved in sensory processing: neurological threshold and behavioural self-regulation response [6]. These refer to the amount of input required for a person to notice sensory stimuli and the indivdual’s response to sensory stimuli (ranging from passive to active), respectively. Some children easily register stimuli (low threshold), whereas others need more input (high threshold) and some children actively seek or avoid stimuli (active self-regulation strategy), while others do not (passive self-regulation strategy). Based on these two dimensions, four sensory processing quadrants can be distinguished: (1) low registration (high threshold, passive strategy), (2) sensation seeking (high threshold, active strategy), (3) sensory sensitivity (low threshold, passive strategy) and (4) sensation avoiding (low threshold, active strategy). Extreme ratings on one or more quadrants indicate sensory processing difficulties. Children with difficulties in the low registration quadrant might, for example, struggle to maintain attention because they need more input to register stimuli [6]. Sensation seeking difficulties may reduce task completion because children get easily distracted by the stimuli they pursue [6]. Sensory sensitivity difficulties may cause distraction or distress because children become overwhelmed by the experienced sensory stimuli [6]. Children with avoiding difficulties might engage less in social situations because they tend to withdraw from stimuli [6].

Previous studies into sensory processing often focused on children with neurodevelopmental disorders, such as autism spectrum disorder (ASD), attention deficit hyperactivity disorder (ADHD) or developmental coordination disorder (DCD). These studies showed that children with these disorders had significantly more impairments across the four sensory processing quadrants than neurotypical children [e.g.,7,8]. For example, they found that children with ASD, ADHD and DCD more often missed, sought, avoided and/or were sensitive to sensory stimuli than typically developing children [7, 8]. This may be explained by altered connectivity within the cerebellum, given its role in sensory processing [3] and in ASD, ADHD and DCD [9, 10]. Additionally, specific sensory processing patterns were related to specific characteristics of ASD, for example, repetitive behaviours were linked to missing sensory stimuli and seeking for them [11].

Nonetheless, sensory processing difficulties may also be present in children without neurodevelopmental disorders. Particularly, Dean et al. [12] showed that 47% of children from the general population experienced sensory processing difficulties in their study, as reflected by extremely low or high scores in (one of) the sensory quadrants. Considering that sensory processing plays an essential role in multiple developmental domains and sensory processing difficulties are generally prevalent, the question arises if sensory processing difficulties in children without a diagnosis of neurodevelopmental disorders are also associated with behavioural difficulties. Previous studies on this topic found that sensory processing difficulties were related to in- and externalising behaviour in 6- to 11-year-old children from the general population [13, 14]. It remains unknown if such associations are also shown in younger children and if sensory processing is related to behavioural characteristics associated with neurodevelopmental disorder, such as ASD, ADHD and DCD. Such an association would be expected based on prior studies on sensory processing in clinical samples, and given that sensory processing plays a role in the diagnostic criteria of ASD [15] and is related to attention [16] and motor behaviour [17].

Therefore, in the current study, by employing a dimensional approach, we examined the relation between scores on the four sensory processing quadrants, as measured with the Sensory Profile [18] and the severity of neurodevelopmental disorder traits, operationalized as scores on instruments measuring ASD, ADHD and DCD behaviours, in a substantial sample of 4- to 5-year-old children from the general population, with an oversampling of children with atypical infant neurodevelopment. To that end, we conducted multivariate polynomial regression analyses, anticipating that both very high and very low scores on the Sensory Profile, each reflecting sensory processing difficulties, would be associated with more severe neurodevelopmental disorder traits. Additionally, we aimed to investigate whether the child’s sex, neurodevelopment in infancy and intelligence level at age 4–5 moderated the association between sensory processing and the severity of neurodevelopmental disorder traits. These variables may be relevant to this association, as previous studies, in the general population have shown that adolescent and adult females are more often hypersensitive to stimuli than males [19], whereas neurodevelopmental disorders show a clear male preponderance [20]. Moreover, because our sample included children with both typical and atypical infant neurodevelopment, we examined whether infant neurodevelopment moderated the associations between sensory processing characteristics and neurodevelopmental disorders traits at age 4–5. Finally, the 4E cognition framework [2], the cerebellar connectome hypothesis [3] and prior research suggest that intelligence should be considered: low intelligence has been associated with sensory processing difficulties [21], and intellectual disabilities and other neurodevelopmental disorders overlap and often co-occur [15].

## Methods

### Participants

The current study is a follow-up in 4- to 5-year-old children from an infant general population cohort. That general population cohort consisted of 1700 infants, between 2 and 18 months old, who were assessed with the Infant Motor Profile (IMP) [22] and the Standardized Infant NeuroDevelopmental Assessment (SINDA) [23]. More information on the original cohort can be found elsewhere [24]. At age 4–5, we invited a part of the children from the general population cohort for a follow-up study, based on their infancy assessment. We invited all children with an atypical infant neurodevelopment (389), as defined by an atypical IMP score (total score < 15th percentile and/or variation or performance domain scores <5th percentile) and/or an atypical SINDA score (neurological score ≤ 21 and/or developmental score ≤ 7). Additionally, 358 randomly selected children with typical infant neurodevelopment (typical IMP and SINDA scores) were invited. Non-responders in the latter group were replaced with a child with typical IMP and SINDA scores, a similar age at the infancy assessment and a similar maternal educational level. The current sample is no longer representative for the general population, due to the oversampling of children with an atypical infant neurodevelopment.

### Procedure

For the follow-up at age 4–5, parents retrieved an information letter about the study including an informed consent form, which they could fill out and send to us. If we did not receive the informed consent form, we contacted the parents after approximately three weeks, to ask if they received the invitation and to ask if they would let their child participate in the study. The children participated in one (or two, if needed based on the child’s pace) video-recorded assessment(s) in our clinic or at home, based on the participants’ preferences. The assessment included the Wechsler Preschool and Primary test of Intelligence (WPPSI-IV-NL) [25] and the Movement Assessment Battery for Children (M-ABC-2) [26]. The assessments were administered by two researchers with an academic background in (child) psychology and by trained students who performed the assessments under supervision. During the assessment, parents completed an online questionnaire that consisted of demographic information and behavioural questionnaires (Sensory Profile [18], Social Responsiveness Scale [27] and Conners Parent Rating Scale [28]). Furthermore, if permitted by the parents, we also invited the child’s teacher to fill out the Strengths and Difficulties Questionnaire [29] and Conners Teacher Rating Scale [30]. The Medical Ethical Committee of the University Medical Center Groningen approved the study (METc 2020/546). We used pseudonymized data, which we store on secure servers of our clinic, and pseudonymized assessments forms which we store in a locked cabinet in a locked room. The study was conducted according to the declaration of Helsinki, the General Data Protection Regulation (GDPR) and Dutch regulations and legislations regarding research.

### Materials

#### Parent-reported measures

*Sensory Profile-Dutch version (SP-NL).* The SP-NL [18] is a parent questionnaire that assesses sensory processing of children between 4 and 12 years. The 125 items have a five-point Likert scale (from 1 ‘Always’ to 5 ‘Never’). For the four sensory processing quadrants, 90 items are used: (1) ‘Low registration’ (the extent to which a child misses stimuli; 15 items), (2) ‘Sensation seeking’ (the extent to which a child pursues stimuli; 26 items), (3) ‘Sensory sensitivity’ (the extent to which a child notices stimuli; 20 items) and (4) ‘Sensation avoiding’ (the extent to which a child is bothered by stimuli and therefore avoids the stimuli; 29 items). The prevalence of sensory processing difficulties was based on the Dutch norm category scores of the four quadrants: (1) ‘less than others’, (2) ‘comparable to others’, (3) ‘more than others’ and (4) ‘much more than others’, with one or two standard deviations from the mean being boundaries for less/more or much less/more, respectively. We could not provide the prevalence of the ‘much less than others’ category, for which unfortunately no Dutch cut offs are available for 4- and 5-year-olds. In the other analyses, we used continuous scores of the sensory quadrants. Lower scores on these quadrants indicate that the measured behaviour in a quadrant is more present in the child, while higher scores indicate that the child shows less of the measured behaviour. The psychometric properties of the Sensory Profile are good (internal consistency: α = .83–.95, test-retest reliability: ICC*=*.80–.90, AUC=.78–.90) [18].

*Social Responsiveness Scale 2 (SRS-2).* The Dutch SRS-2 [27] is a screening questionnaire for ASD in children between 4 and 18 years. The 65 items are rated on a four-point Likert scale (from 0 ‘Not true’ to 3 ‘Almost always true’), except for 17 items in which the scoring is reversed. Adding all item scores yields the total score, with higher scores indicating more severe ASD traits. For the Dutch general population, the cut-off score for elevated risk for ASD is > 51. Studies into the psychometric qualities showed good internal consistency (α = 0.93–0.95) and high predictive values for an ASD diagnosis (sensitivity=0.90 and specificity=0.88) [27].

*Conners’ parent rating scale revised long version (CPRS-R).* The CPRS-R [28], completed by parents, is an 80-item screening questionnaire for behavioural problems, including ADHD. The items are answered on a four-point Likert scale (from 0 ‘Not at all true’ to 3 ‘Very much true’). We solely focused on the subscale ‘Diagnostic and Statistical Manual of Mental Disorders (DSM) total ADHD’, which combines 18 items on inattention and hyperactivity. Higher scores indicate more severe ADHD traits. A percentile score of 93.3 indicates an elevated risk of ADHD [31]. Research into the psychometric properties showed high internal consistencies (α = 0.77–0.92) and predictive values for an ADHD diagnosis (sensitivity=0.92, specificity=0.94) [28].

#### Teacher-reported measures

*Strengths and difficulties questionnaire (SDQ).* The Dutch version of the teacher SDQ is a screening questionnaire for behavioural strengths and difficulties in children between 3 and 17 years old [29]. It has 25 items with a three-point Likert scale (from 0 ‘Not true’ to 2 ‘Definitely true’). The five subscales consist of five items each: (1) ‘Emotional symptoms’, (2) ‘Conduct problems’, (3) ‘Hyperactivity/inattention’, (4) ‘Peer relationship problems’ and (5) ’Prosocial behaviour’. We used scores on the subscales ‘Peer relationship problems’ and ‘Prosocial behaviour’ as a teacher-based proxy for the severity of ASD traits as suggested previously [32]. The scoring of the prosocial behaviour scale items was reversed and a mean score for both subscales was calculated. Higher scores indicate more severe ASD traits. Previous studies showed satisfactory (α = .64–.67) and good (α = .81–.82) internal consistencies for peer relationship problems and prosocial behaviour, respectively and a reliable test-retest correlation (*r=.*65 and *r=*.70, respectively*)* [33].

*Conners’ teacher rating scale revised short version (CTRS-R).* The CTRS-R [30] assesses the child’s behaviour in the classroom based on the teacher’s perception. The 28 items are answered on a four-point Likert scale (from 0 ‘Not true at all’ to 3 ‘Very much true’). In the current study, the ADHD index subscale was used to assess the severity of ADHD traits. Higher scores indicate more severe ADHD traits. A percentile score of 93.3 indicates an elevated risk of ADHD [31]. The CTRS-R is internally consistent (α = 0.73–0.95) and has satisfactory predictive values for ADHD (sensitivity=0.72 and specificity=0.84) [30, 34].

#### Child measures

*Movement Assessment Battery for Children 2.* The Dutch M-ABC-2 is a clinical instrument that assesses motor development in children between 3 and 16 years [26]. We used the first age band, for 3-6-year-olds, which contains eight test items, administered in about 30 min, providing information on three motor domains: manual dexterity (3 items), aiming and catching (2 items) and balance (3 items). We used the total score in the main analyses; which we reverse-coded such that higher scores indicate more severe DCD traits and could be more easily compared to the scores of the other neurodevelopmental disorder traits. The cut-off score for risk of DCD is a percentile score of 5 or lower. The M-ABC-2 has a high internal consistency (α = 0.83) and test-retest reliability (ICC=0.83) [35].

*Wechsler Preschool and Primary Scale of Intelligence IV (WPPSI-IV-NL*). To assess intelligence at age 4–5, the Dutch version of the WPPSI-IV was used which is applicable for children between 2:6 and 6:11 years old [25]. We used a continuous total intelligent quotient (TIQ) in the main analyses. For descriptive purposes, we also dichotomized the TIQ: low intelligence level (TIQ < 70) and average to high intelligence level (TIQ ≥ 70).

*Other materials.* Demographic variables (e.g., the child’s sex, parental ethnicity; requested by the open-ended question ‘ethnical background mother: …’ and ‘ethnical background father: …’), parental educational level and perinatal background variables were collected through a parental questionnaire during the original study. In the follow-up, parental educational level was requested again by means of a questionnaire.

### Statistical analyses

We used descriptive statistics to describe our study sample on various background variables, sensory processing scores and severity ratings of neurodevelopmental disorder traits (ASD traits as measured with the SRS-2 and SDQ; ADHD traits as measured with the CPRS-R and CTRS-R; DCD traits as measured with the M-ABC-2) for the total group, and disaggregated by infant neurodevelopmental status (typical/atypical). Additionally, we compared our sample and the non-responders with independent sample t-tests and chi-square tests. Furthermore, we examined the prevalence of sensory processing difficulties based on the frequencies of the Sensory Profile quadrant categories (‘less than others’, ‘comparable to others’, ‘more than others’ and ‘a lot more than others’). We conducted multivariate polynomial regression analyses to investigate the association between sensory processing and the severity of neurodevelopmental disorder traits, and to test whether this association was moderated by the child’s sex, infant neurodevelopment status and intelligence at school-age. We also included quadratic sensory processing terms, as both very high and very low scores may reflect sensory processing difficulties. We first completed two multivariate polynomial regression analyses in which we included the four continuous sensory processing quadrant scores (centred), the quadratic terms of these scores, the moderators (the child’s sex (boy/girl), infant neurodevelopmental status (typical/atypical) and intelligence score (centred)) and their interaction terms as predictor variables. One model was performed with the severity of observed DCD traits, parent-reported ASD traits and parent-reported ADHD traits, and one model included the severity of observed DCD traits, teacher-reported ASD and teacher-reported ADHD traits as outcome variables. Second, we repeated the two multivariate polynomial regression analyses yet excluding possible nonsignificant interaction terms of the moderator variables. Third, we repeated the two multivariate polynomial regression analyses again without possible nonsignificant quadratic terms of the sensory processing scores. An alpha level of .05 was considered statistically significant and a Hochberg procedure was performed to control for multiple comparisons. We used Pillai’s trace and robust standard errors to ensure reliable test statistics in the multivariate and coefficient tests. Furthermore, partial eta squared was used to indicate a small (.01), medium (.06) or large (.14) effect [36]. The analyses were performed in SPSS (version 28.0.1.0) and in R Studio (2025.05.1).

## Results

### Participants

Of the invited children, 557 (74%) participated in the current study. There were no significant differences in the child’s sex, gestational age, birth weight and maternal ethnicity between the included sample and the non-responders. However, the included sample contained significantly fewer children with an atypical infant neurodevelopment than the non-responders (44% vs. 80%, χ2 = 73.108, *p*<.001). Additionally, in the current sample, there were significantly more mothers and fathers with a high educational level (45% vs. 28%, χ2 = 28.109, *p*<.001 and 42% vs. 24%, χ2 = 37.027, *p*<.001, respectively) and more fathers with a Dutch descent (94% vs. 85%, χ2 = 19.202, *p*=.004) in comparison to the non-responders. The parental questionnaires were completed by 536 (97%) parents, the teacher questionnaires by 296 (54%) teachers and the M-ABC-2 and WPPSI-IV-NL were administered in 551 and 554 (99%) children, respectively. Table 1 shows the demographic characteristics of the sample and mean scores on the dependent and independent variables. The prevalences of the sensory processing patterns were: 16–24% for the ‘less than others’ pattern, 56–65% for the ‘comparable to others’ pattern, 9–12% for the ‘more than others’ pattern and 5–8% for the ‘much more than others’ pattern. Furthermore, 16% of the children scored above the cut-off on parent-reported ASD traits, 9% on parent-reported ADHD traits, 3% on teacher-reported ADHD traits, 2% and 26% below the cut-off on IQ and DCD traits, respectively. Supplementary Table S1a displays the prevalence of the four sensory processing quadrants categories, as well as the percentages of children who scored above the cut-off on parent-reported ASD and ADHD traits, teacher-reported ADHD traits and below the cut-off on IQ and DCD traits, by infant neurodevelopmental status (typical/atypical). In Supplementary Table S1b, the percentages of children with scores below or above the cut-off on the neurodevelopmental disorder traits are presented by the sensory processing quadrant categories.

**Table 1:** Demographic information and scores on the outcome measurements (*n* = 557).

Age (months): median (range)	64 (58–71)
Sex: boy/girl; *n* (%)	290 (52%), 267 (48%)
IMP/SINDA score: typical/atypical infant neurodevelopment	240 (43%), 317 (57%)
Maternal educational level: low/middle/high^a^; *n* (%)	46 (8%), 248 (45%), 244 (44%)
Maternal ethnicity: Dutch/non-Dutch; *n* (%)	508 (91%), 49 (9%)
Paternal educational level: low/middle/high^a^, *n* (%)	52 (9%), 253 (45%), 225 (40%)
Paternal ethnicity, Dutch/non-Dutch, *n* (%)	513 (92%), 28 (8%)
Gestational age (weeks): median (range)	39.6 (27. 39–42.1)
Small for gestational age: yes/no; *n (%)*	58 (10%), 498 (89%)
Birth weight (grams): *M* (*SD*)	3431.8 (601.7)
SP-NL; Low registration: **1**/2/**3**/**4**^b^; *n* (%)	**90 (16%)**/ 361 (65%)/ **48 (9%)**/ **43 (8%)**
SP-NL; Sensation seeking: **1**/2/**3**/**4**^b^; *n* (%)	**130 (23%)**/ 319 (57%)/ **67 (12%)**/ **26 (5%)**
SP-NL; Sensory sensitivity: **1**/2/**3**/**4**^b^; *n* (%)	**130 (23%)**/ 309 (56%)/ **61(11%)**/ **42 (8%)**
SP-NL; Sensation avoiding: **1**/2/**3**/**4**^b^; *n* (%)	**135 (24%)**/ 333 (60%)/ **48 (9%)**/ **26 (5%)**
SP-NL; Low registration: *M (SD)*	67.7 (7.2)
SP-NL; Sensation seeking: *M (SD)*	109.3 (14.3)
SP-NL; Sensory sensitivity: *M (SD)*	87.8 (10.0)
SP-NL; Sensation avoiding: *M (SD)*	124.6 (13.1)
SRS total score: *M (SD)*	34.6 (20.7)
SDQ autism score: *M (SD)*	1.8 (1.6)
CPRS-R ADHD DSM-T^c^ score: *M (SD)*	11.4 (8.8)
CTRS-R ADHD index score: *M (SD)*	6.4 (7.0)
M-ABC-2 total score: *M* (*SD*)	7.52 (2.90)
WPPSI-IV-NL *M (SD)*	97.7 (14.0)

Note. *IMP =* Infant Motor Profile, *SINDA* = Standardized Infant NeuroDevelopmental Assessment, *SP-NL* = Sensory Profile, *SRS* = Social Responsiveness Scale, *SDQ =* Strengths and Difficulties Questionnaire, *CPRS-R* = Conners Parent Rating Scale Revised, *CTRS-R* = Conners Teacher Rating Scale Revised, *WPPSI-IV-NL* = Dutch Wechsler Preschool and Primary Scale of Intelligence IV, *M-ABC-2* = Movement Assessment Battery for Children second edition

^a^ low: no or primary school, pre-vocational secondary education, or secondary vocational education level 1, middle: senior general secondary education, pre-university education or vocational secondary education level 2–4, high: higher professional education and university education

^b^ 1: less than others, 2: comparable to others, 3: more than others, 4: much more than others, the bold numbers refer to sensory processing difficulties

^c^ Diagnostic and Statistical Manual total scale

### The relation between sensory processing and the severity of neurodevelopmental traits

The first two multivariate polynomial regression analyses, including the quadratic sensory processing terms, the centred sensory processing variables, the moderating variables and their interaction terms, showed that the relation between sensory processing and the severity of neurodevelopmental disorder traits was not moderated by sex, infant neurodevelopmental status and intelligence score (see Supplementary Table S2 for the results of the multivariate effects). Therefore, we removed the non-significant interaction effects from the models and only included the main effects of the moderators and the quadratic and centred sensory processing variables. In the new model with the parent-reported neurodevelopmental disorder traits, the quadratic terms of the sensory sensitivity and sensation avoiding quadrants were nonsignificant and in the new model with the teacher-reported neurodevelopmental disorder traits, none of the quadratic sensory processing quadrant scores were significant (see Supplementary Table S3).

To enhance model parsimony and interpretation of the results, we ran two final multivariate regression models, in which we only included the significant quadratic sensory processing terms of the former model, the centred sensory processing variables and the covariates (the child’s sex, infant neurodevelopmental status and intelligence score). Supplementary Table S4 presents the results of these final multivariate regression models. Below, we discuss the most important findings. In the parent-reported model, we found that low registration, sensation seeking, sensation avoiding and sensory sensitivity were linearly associated with the severity of neurodevelopmental disorder traits (Pillai’s Trace=0.022-0.091, *F*(3,524) = 3.88–17.4, *p*<.001–*p*=.009). As shown in Table 2, lower low registration and sensory sensitivity scores were related to more severe ASD, ADHD and DCD traits. Furthermore, lower sensation seeking scores were related to more severe ASD and ADHD traits and to less severe DCD traits. Additionally, sensation avoiding was associated with more severe ASD traits. Moreover, low registration and sensation seeking were nonlinearly related to the severity of neurodevelopmental disorder traits (Pillai’s Trace=0.042–0.054, *F*(3,524) = 7.64–10.0, *p*<.001, *ηp²*=0.042–0.054). Particularly, we found that low registration had a negative nonlinear relation with ADHD and DCD traits (See Table 2). Similarly, a positive, nonlinear relation was found between sensation seeking and ASD and ADHD traits. Supplementary Fig. 1 can be used to interpretate these nonlinear findings. The teacher-reported model only revealed a linear relation between sensation seeking and the severity of neurodevelopmental disorder traits (Pillai’s Trace=0.047, *F*(3,286) = 4.70, *p*=.003, *ηp²*=0.047). As presented in Table 2, lower sensation seeking scores were related to more severe ADHD traits.

**Table 2:** Significant associations between sensory processing and neurodevelopmental disorder traits

Model with parent-reported ASD/ADHD traits (*n* = 536)							
		*b* *(SE*)			95%* CI*	*ηp²*	
Low registration							
	ASD traits	− 0.58 (0.17)			[-0.89, − 0.26]	0.024	
	ADHD traits	− 0.37 (0.075)			[-0.52, − 0.23]	0.047	
	DCD traits	− 0.13 (0.032)			[-0.20, − 0.067]	0.030	
Sensation seeking							
	ASD traits	− 0.19 (0.065)			[-0.32, − 0.067]	0.017	
	ADHD traits	− 0.19 (0.028)			[-0.25, − 0.13]	0.073	
	DCD traits	0.030 (0.013)			[0.0041, 0.055]	0.010	
Sensory sensitivity							
	ASD traits	− 0.36 (0.12)			[-0.56, − 0.15]	0.022	
	ADHD traits	− 0.19 (0.048)			[-0.29, − 0.097]	0.029	
	DCD traits	− 0.044 (0.022)			[-0.086, − 0.0023]	0.0081	
Sensation avoiding							
	ASD traits	− 0.22 (0.095)			[-0.38, − 0.068]	0.015	
Low registration (quadratic)							
	ADHD traits	− 0.010 (0.0032)			[-0.016, − 0.0042]	0.021	
	DCD traits	− 0.0044 (0.0015)			[-0.0070, − 0.0017]	0.020	
Sensation seeking (quadratic)							
	ASD traits	0.0063 (0.0038)			[0.0022, 0.010]	0.017	
	ADHD traits	0.0042 (0.0010)			[0.0023, 0.0061]	0.034	
Model with teacher-reported ASD/ADHD traits (*n* = 296)							
		*b* (*SE*)			95% *CI*	*ηp²*	
Sensation seeking							
	ADHD traits	− 0.13 (0.048)			[-0.21, − 0.054]	0.037	

Note. *ASD* = Autism Spectrum Disorder, *ADHD* = Attention Deficit Hyperactivity Disorder, *DCD* = Developmental Coordination Disorder

Lower scores on sensory quadrants indicate more sensory-related behaviour. For all neurodevelopmental disorder traits, higher valuesindicate more severe difficulties

## Discussion

Sensory processing difficulties were relatively prevalent (35–44%) in a sample of 4- to 5-year-old children, enriched for the presence of atypical infant neurodevelopment. Furthermore, we found a relation between sensory processing and the severity of neurodevelopmental disorder traits (especially as reported by parents). Particularly, registering little sensory stimuli was linearly related to more severe ASD, ADHD and DCD traits. However, the small negative nonlinear relation between low registration and ADHD and DCD traits in our sample, indicate that registering many sensory stimuli is also associated with more severe ADHD and DCD traits. Seeking sensory stimuli was related to more severe ASD and ADHD traits and less severe DCD traits. Again, a small positive curvature in the association between sensation seeking with ASD and ADHD traits indicates that not seeking sensory stimuli was also related to more severe ASD and ADHD traits. Being sensitive for sensory stimuli was associated with more severe ASD, ADHD and DCD traits, while avoiding sensory stimuli was solely related to more severe ASD traits.

The mean sensory processing quadrant scores in our sample are comparable to those of the Dutch norm group of the SP-NL (except for sensation avoiding, as shown in Supplementary Table S5) [18]. Similarly, the prevalence of sensory processing difficulties in the current study corresponds to that of previous studies in general populations [12, 37]. This indicates that sensory processing difficulties are present in a broader group of children than only in those with neurodevelopmental disorders. Although the sensory processing patterns of these children are different from the most common sensory processing patterns, children may only experience difficulties if their sensory processing patterns affect their participation in daily life [6]. A child with sensory sensitivities, for example, might have trouble completing tasks at school. However, when the environment is tailored to the child’s needs, for example, by using headphones in a loud classroom, a child with sensory sensitivities may be able to adequately focus on a task.

In concordance with the 4E cognition framework [2] and the cerebellar connectome hypothesis [3], we found that missing, seeking, avoiding or being sensitive to sensory stimuli were associated with more severe neurodevelopmental disorder traits. This finding is in line with prior studies that were conducted in children in clinical samples [e.g., 38] and adults of the general population [e.g., 39], which found that sensory processing difficulties were related to behavioural difficulties such as inattention, hyperactivity, in- and externalising problems. Similarly, in a previous study in children from the general population, sensory processing difficulties were related to behavioural difficulties (i.e., depressive symptoms and externalising problems among which hyperactivity) [13].

Our findings that sensation avoiding was related to ASD but not to ADHD traits corroborates previous studies that showed that children with ASD more often miss, seek, avoid and/or are sensitive to sensory stimuli, while children with ADHD did not avoid sensory stimuli [8]. Children with ADHD are often more prone to hypoarousal and therefore may more often seek stimuli rather than avoid them [40]. However, other studies found that children with ADHD showed more avoiding behaviour towards sensory stimuli [41, 42], which underlines the need for a dimensional approach in research on neurodevelopmental disorders due to the heterogeneity between children within the same neurodevelopmental disorder and behavioural overlap between distinct neurodevelopmental disorders [43]. We did not find a relation between sensation avoiding and the severity of DCD traits, while in a previous study children with DCD or at risk of DCD more often avoided sensory stimuli than typically developing children [7, 44] The difference between our study and these previous studies is that we did not compare clinical groups but dimensionally addressed both sensory processing and the severity of neurodevelopmental disorder traits, aiming to capture more nuanced variation in behaviour [45]. Moreover, we found that, while simultaneously including ASD and ADHD in the statistical model, sensory processing and DCD traits were significantly related. This contrasts with a previous study, performed in 8-12-year-olds, which found that motor ability was not significantly related to sensory processing when parent-reported ASD and ADHD traits were concurrently included in the statistical model [46]. This indicates that motor behaviour and sensory processing are more closely related at a younger age than at a later age. Last, in contrast to our hypothesis, seeking sensory stimuli was related to less severe DCD traits. A potential explanation for this finding is that children who more often seek sensory stimuli might also explore more. This will provide children with more trial-and-error experiences, which are important to enhance their motor development [17].

Our findings did not completely align with the conceptual framework of the Sensory Profile, in which both children with very high and very low scores on the Sensory Profile may experience sensory processing difficulties [18]. We only found small nonlinear relations between low registration and sensations seeking quadrants and some of the neurodevelopmental disorder traits. We found more pronounced linear associations across all sensory quadrants, which indicates that a specific sensory processing pattern, i.e., missing, seeking, avoiding and/or being sensitive to sensory stimuli is related to less social responsiveness, more hyperactivity and/or inattentive behaviour and more motor difficulties. We found, for example, that more actively seeking sensory stimuli is related to more hyperactive and/or inattentive behaviour. In educational contexts, it might be difficult for these children to concentrate on tasks. Knowing how children process sensory information and what kind of behaviour is related to this sensory processing, helps to accommodate their sensory preferences and their needs for school tasks. A reason why children with other sensory processing patterns (i.e., who register all stimuli, who do not seek, who do not avoid or who are not sensitive to sensory stimuli) might have less severe neurodevelopmental disorder traits, is that these children may process information in a more structured and less sensory-driven manner, which could explain why they may be less susceptible to behaviours associated with neurodevelopmental conditions.

Notably, the relations between sensory processing and the severity of neurodevelopmental disorder traits were mostly found in the parent-reported models and only for sensation avoiding and ASD traits in the teacher-reported models. Parents also evaluated their child’s behaviour more often as indicative of ASD and ADHD traits than teachers. This discrepancy in parent and teacher reports has been reported previously [e.g., 47]. Although this difference may partially be explained by same informant bias, as parents rated both sensory processing and the severity of ASD and ADHD traits, also the context in which the behaviour of the child is rated may form a likely explanation [48]. Particularly, the expectations and demands may differ in various contexts, which could affect the child’s behaviour and the informant’s perception of it.

A strength of the current study was the use of a multi-informant approach by evaluating the child’s behaviour in various contexts. Furthermore, we took into account children’s intelligence scores while examining the relation between sensory processing and the severity of neurodevelopmental traits, which is important, given the large effect sizes of intelligence in the models. Additionally, we used a dimensional approach to address the severity of neurodevelopmental disorder traits by examining several traits of different neurodevelopmental disorders without using diagnoses. Our study has some limitations too. First, the generalisability of the results to the general population is limited as our sample consists of 4- to 5-year-old children, enriched for the presence of atypical infant neurodevelopment. However, the non-significant interaction and main effects of infant neurodevelopmental status indicates that infant neurodevelopment does not play a moderating role in the relation between sensory processing and severity of neurodevelopmental disorder traits nor affected the outcome variables. Second, data from the teacher reports were frequently missing, preventing us from adding the teacher- and parent-reported variables in one model. Third, as we used a version of the CPRS-R and CTRS-R that was not validated in the Dutch population, the original American norms were used in the current study, which may affect the reliability and validity. However, we only used the cut-off scores for descriptive purposes, while in the regression analyses, we used the continuous scores. Similarly, as for the Dutch population no cut-offs for the sensory processing category ‘much less than others’ are available, we unfortunately could not examine the prevalence of this group. Fourth, most data were collected with questionnaires, perhaps affecting the objectivity of the measured constructs and potentially overestimating the presence of behaviours related to neurodevelopmental disorders [49], especially in the parent-reported models in which the parents reported on both sensory processing behaviour and the severity of neurodevelopmental disorder traits. Lastly, prior studies showed that sensory processing difficulties were occurring more frequently in children with sleep problems than in children without sleep problems [50]. This suggests that sleep might be a confounding variable in the current study. Unfortunately, we could not account for sleep problems in our analyses and therefore, we recommend future studies on sensory processing to include data on sleep behaviour. Furthermore, future research in population-based samples, may examine if and how sensory processing difficulties affect a child’s daily life, and if and what support is needed to enhance participation.

In conclusion, sensory processing difficulties are quite prevalent in a sample of 4- to 5-year-old children, enriched for the presence of atypical infant neurodevelopment and are not restricted to children with a diagnosis of neurodevelopmental disorders. Notably, in this broad sample specific sensory processing difficulties (i.e., missing, seeking, avoiding and/or being sensitive to sensory stimuli) were related to less social responsiveness, more hyperactive and/or inattentive behaviour and more motor difficulties. The current findings imply that identifying and supporting sensory processing needs in all children is essential to stimulate the child’s development and to promote participation in daily life activities.

## Supplementary information

Below is the link to the electronic supplementary material.

Supplementary Material 1

## Data Availability

The datasets generated and analysed during the current study are not publicly available due to privacy and ethical restrictions but are available from the corresponding author on reasonable request.
