# Supplementary material for: Sensory processing and neurodevelopmental disorder traits in 4- to 5-year-old Dutch children adjusted for sex, neurodevelopment in infancy and IQ
Source: Eur Child Adolesc Psychiatry. 2026 Jul 6;35(11):3549–58. doi: 10.1007/s00787-026-03110-7 (PMC13710646; doi:10.1007/s00787-026-03110-7)
Supplement: Supplementary file 1 [file 787_2026_3110_MOESM1_ESM.docx]

Title: Sensory processing and neurodevelopmental disorder traits in 4- to 5-year-old Dutch children adjusted for sex, neurodevelopment in infancy and IQ

Journal name: European Child and Adolescence Psychiatry

Author names: Selena J Rosinda, Pieter J Hoekstra, Mijna Hadders-Algra, Kirsten R. Heineman, Annelies de Bildt

Corresponding author information: [s.j.rosinda@umcg.nl](mailto:s.j.rosinda@umcg.nl) | University of Groningen, University Medical Center Groningen, Department of Paediatrics, Beatrix Children’s Hospital, Division of Developmental Neurology, Groningen, the Netherlands | Accare Child Study Center, Groningen, the Netherlands | The Research School of Behavioural and Cognitive Neurosciences, University of Groningen, Groningen, the Netherlands

Supplementary Figure 1. Plots significant nonlinear regression lines.

Supplementary Table S1a. *Scores sensory processing and neurodevelopmental disorder traits for the children with a typical and atypical infant neurodevelopment.*

|  | Typical infant neurodevelopment  (*n* = 317) | Atypical infant neurodevelopment  (*n* = 240) |
| --- | --- | --- |
| SP-NL; Low registration: 1/2/3/4^a^; *n* (%) | 54 (17%), 213 (67%), 29 (9%), 14 (4%) | 36 (15%), 148 (62%), 19 (8%), 29 (12%) |
| SP-NL; Sensation seeking: 1/2/3/4^a^; *n* (%) | 72 (23%), 186 (59%), 39 (12%), 13 (4%) | 58 (24%), 133 (55%), 28 (12%), 13 (5%) |
| SP-NL; Sensory sensitivity: 1/2/3/4^a^; *n* (%) | 73 (23%), 184 (58%), 34 (11%), 19 (6%) | 57 (24%), 125 (52%), 27 (11%), 23 (10%) |
| SP-NL; Sensation avoiding: 1/2/3/4^a^; *n* (%) | 79 (25%), 190 (60%), 28 (9%), 13 (4%) | 56 (23%), 143 (60%), 20 (8%), 13 (5%) |
| SRS-2; above the cut-off: *n* (%) | 44 (14%) | 43 (18%) |
| CPRS-R; above the cut-off: *n* (%) | 31 (10%) | 20 (8%) |
| SDQ | * | * |
| CTRS-R; above the cut-off: *n* (%) | 8 (3%) | 7 (3%) |
| M-ABC-2; below the cut-off: *n* (%) | 73 (23%) | 72 (30%) |
| SRS-2 and CPRS-R above the cut-off: *n* (%) | 13 (4%) | 15 (6%) |
| SRS-2 above and M-ABC-2 below the cut-off: *n* (%) | 15 (5%) | 23 (10%) |
| CPRS-R above and M-ABC-2 below the cut-off: *n* (%) | 10 (3%) | 10 (4%) |
| SRS-2, CPRS-R above and M-ABC-2 below the cut-off: *n* (%) | 0 (0%) | 3 (1%) |
| WPPSI-IV-NL; below the cut-off: *n* (%) | 2 (1%) | 8 (3%) |

*Note.* SP-NL = Sensory Profile, SRS-2 = Social Responsiveness Scale, SDQ = Strengths and Difficulties Questionnaire, CPRS-R = Conners Parent Rating Scale Revised, CTRS-R = Conners Teacher Rating Scale Revised, WPPSI-IV-NL = Wechsler Preschool and Primary Scale of Intelligence, M-ABC-2 = Movement Assessment Battery for Children second edition.

*^a^* 1: less than others, 2: comparable to others, 3: more than others, 4: much more than others

* No cut-off scores are available for the SDQ teacher-based proxy for the severity of ASD traits

Supplementary Table 1b. *Scores severity of neurodevelopmental disorder traits for children with different sensory processing patterns, per quadrant.*

| **Low registration** |  |  |  |  |
| --- | --- | --- | --- | --- |
|  | Less  than others  (*n* = 90) | Comparable  to others  (*n* = 361) | More  than others  (*n* = 48) | Much more than others  (*n* = 43) |
| SRS-2; above the cut-off: *n* (%) | 0 (0%) | 36 (10%) | 15 (31%) | 29 (67%) |
| CPRS-R; above the cut-off: *n* (%) | 1 (1%) | 17 (5%) | 14 (29%) | 17 (40%) |
| SDQ | * | * | * | * |
| CTRS-R; above the cut-off: *n* (%) | 0 (0%) | 10 (3%) | 4 (8%) | 1 (2%) |
| M-ABC-2; below the cut-off: *n* (%) | 13 (14%) | 87 (24%) | 21 (44%) | 19 (44%) |
| SRS-2 and CPRS-R above the cut-off: *n* (%) | 0 (0%) | 2 (1%) | 8 (17%) | 16 (37%) |
| SRS-2 above and M-ABC-2 below the cut-off: *n* (%) | 0 (0%) | 12 (3%) | 9 (19%) | 14 (33%) |
| CPRS-R above and M-ABC-2 below the cut-off: *n* (%) | 1 (1%) | 6 (2%) | 6 (13%) | 7 (16%) |
| SRS-2, CPRS-R above and M-ABC-2 below the cut-off: *n* (%) | 0 (0%) | 1 (0.3%) | 2 (4%) | 0 (0%) |
| WPPSI-IV-NL; below the cut-off: *n* (%) | 1 (1%) | 5 (1%) | 0 (0%) | 3 (7%) |
| **Sensation seeking** |  |  |  |  |
|  | Less  than others  (*n* = 130) | Comparable  to others  (*n* = 319) | More  than others  (*n* = 67) | Much more than others  (*n* = 26) |
| SRS-2; above the cut-off: *n* (%) | 2 (2%) | 36 (11%) | 23 (34%) | 19 (73%) |
| CPRS-R; above the cut-off: *n* (%) | 2 (2%) | 18 (6%) | 14 (21%) | 15 (58%) |
| SDQ | * | * | * | * |
| CTRS-R; above the cut-off: *n* (%) | 0 (0%) | 9 (3%) | 4 (6%) | 2 (8%) |
| M-ABC-2; below the cut-off: *n* (%) | 33 (25%) | 69 (22%) | 22 (33%) | 16 (62%) |
| SRS-2 and CPRS-R above the cut-off: *n* (%) | 0 (0%) | 7 (2%) | 7 (10%) | 12 (46%) |
| SRS-2 above and M-ABC-2 below the cut-off: *n* (%) | 1 (1%) | 11 (3%) | 12 (18%) | 11 (42%) |
| CPRS-R above and M-ABC-2 below the cut-off: *n* (%) | 0 (0%) | 8 (3%) | 3 (5%) | 9 (35%) |
| SRS-2, CPRS-R above and M-ABC-2 below the cut-off: *n* (%) | 0 (0%) | 1 (0.3%) | 2 (3%) | 0 (0%) |
| WPPSI-IV-NL; below the cut-off: *n* (%) | 1 (1%) | 4 (1%) | 3 (5%) | 1 (4%) |
| **Sensory sensitivity** |  |  |  |  |
|  | Less  than others  (*n* = 130) | Comparable  to others  (*n* = 309) | More  than others  (*n* = 61) | Much more than others  (*n* = 42) |
| SRS-2; above the cut-off: *n* (%) | 2 (2%) | 30 (10%) | 18 (30%) | 30 (71%) |
| CPRS-R; above the cut-off: *n* (%) | 1 (1%) | 23 (7%) | 6 (10%) | 19 (45%) |
| SDQ | * | * | * | * |
| CTRS-R; above the cut-off: *n* (%) | 0 (%) | 9 (3%) | 3 (5%) | 3 (7%) |
| M-ABC-2; below the cut-off: *n* (%) | 27 (21%) | 69 (22%) | 23 (38%) | 21 (50%) |
| SRS-2 and CPRS-R above the cut-off: *n* (%) | 0 (0%) | 4 (1%) | 5 (8%) | 17 (41%) |
| SRS-2 above and M-ABC-2 below the cut-off: *n* (%) | 1 (1%) | 10 (3%) | 8 (13%) | 16 (38%) |
| CPRS-R above and M-ABC-2 below the cut-off: *n* (%) | 0 (0%) | 8 (3%) | 2 (3%) | 10 (24%) |
| SRS-2, CPRS-R above and M-ABC-2 below the cut-off: *n* (%) | 0 (0%) | 1 (0.3%) | 0 (0%) | 2 (5%) |
| WPPSI-IV-NL; below the cut-off: *n* (%) | 3 (2%) | 3 (1%) | 0 (0%) | 3 (7%) |
| **Sensation avoiding** |  |  |  |  |
|  | Less  than others  (*n* = 135) | Comparable  to others  (*n* = 333) | More  than others  (*n* = 48) | Much more than others  (*n* = 26) |
| SRS-2; above the cut-off: *n* (%) | 3 (2%) | 38 (11%) | 16 (33%) | 23 (89%) |
| CPRS-R; above the cut-off: *n* (%) | 4 (3%) | 23 (7%) | 8 (17%) | 14 (54%) |
| SDQ | * | * | * | * |
| CTRS-R; above the cut-off: *n* (%) | 1 (1%) | 12 (4%) | 0 (0%) | 2 (8%) |
| M-ABC-2; below the cut-off: *n* (%) | 27 (20%) | 85 (26%) | 16 (33%) | 12 (46%) |
| SRS-2 and CPRS-R above the cut-off: *n* (%) | 0 (0%) | 9 (3%) | 4 (8%) | 13 (50%) |
| SRS-2 above and M-ABC-2 below the cut-off: *n* (%) | 0 (0%) | 17 (5%) | 6 (13%) | 10 (38%) |
| CPRS-R above and M-ABC-2 below the cut-off: *n* (%) | 1 (1%) | 11 (3%) | 3 (6%) | 5 (19%) |
| SRS-2, CPRS-R above and M-ABC-2 below the cut-off: *n* (%) | 0 (0%) | 2 (1%) | 0 (0%) | 1 (4%) |
| WPPSI-IV-NL; below the cut-off: *n* (%) | 1 (1%) | 5 (15%) | 0 (0%) | 3 (12%) |

*Note.* SP-NL = Sensory Profile, SRS-2 = Social Responsiveness Scale, SDQ = Strengths and Difficulties Questionnaire, CPRS-R = Conners Parent Rating Scale Revised, CTRS-R = Conners Teacher Rating Scale Revised, WPPSI-IV-NL = Wechsler Preschool and Primary Scale of Intelligence, M-ABC-2 = Movement Assessment Battery for Children second edition. * No cut-off scores are available for the SDQ teacher-based proxy for the severity of ASD traits

Table S2. *Multivariate effects of the initial multivariate polynomial regression models examining the relation between sensory processing and neurodevelopmental traits including the moderating role of the child’s sex (boy/girl), infant neurodevelopment (typical/atypical) and intelligence level (continuous) at 4-5 years.*

| Parent-reported model (*n*=536) | | | | |
| --- | --- | --- | --- | --- |
|  | *Pillai’s Trace* | *F(3,524)* | *p* | *ηp²* |
| Low registration | .0035 | .560 | .623 | .0035 |
| Sensation seeking | .015 | 2.5 | .063 | .015 |
| Sensory sensitivity | .011 | 1.8 | .149 | .011 |
| Sensation avoiding | .0084 | 1.4 | .240 | .0084 |
| Low registration quadratic term | .0046 | .77 | .512 | .0046 |
| Sensation seeking quadratic term | .0040 | .66 | .576 | .0040 |
| Sensory sensitivity quadratic term | .0054 | .90 | .441 | .0054 |
| Sensation avoiding quadratic term | .0088 | 1.5 | .222 | .0088 |
| Sex | .010 | 1.7 | .171 | .010 |
| Infant neurodevelopment | .0067 | 1.1 | .339 | .0067 |
| Intelligence | .10 | 19 | **<.001** | .10 |
| Low registration x Sex | .0038 | .63 | .599 | .0038 |
| Sensation seeking x Sex | .0023 | .39 | .762 | .0023 |
| Sensory sensitivity x Sex | .0053 | .89 | .446 | .0053 |
| Sensation avoiding x Sex | .0055 | .92 | .430 | .0055 |
| Low registration quadratic term x Sex | .0039 | .65 | .582 | .0039 |
| Sensation seeking quadratic term x Sex | .0022 | .37 | .777 | .0022 |
| Sensory sensitivity quadratic term x Sex | .0021 | .35 | .787 | .0021 |
| Sensation avoiding quadratic term x Sex | .0024 | .40 | .755 | .0024 |
| Low registration x Infant neurodevelopment | .0012 | .21 | .893 | .0012 |
| Sensation seeking x Infant neurodevelopment | .0088 | 1.5 | .220 | .0088 |
| Sensory sensitivity x Infant neurodevelopment | .0045 | .76 | .518 | .0045 |
| Sensation avoiding x Infant neurodevelopment | .0034 | .57 | .634 | .0034 |
| Low registration quadratic term x Infant neurodevelopment | .0076 | 1.3 | .285 | .0076 |
| Sensation seeking quadratic term x Infant neurodevelopment | .010 | 1.7 | .164 | .010 |
| Sensory sensitivity quadratic term x Infant neurodevelopment | .0043 | .71 | .546 | .0043 |
| Sensation avoiding quadratic term x Infant neurodevelopment | .0044 | .74 | .531 | .0044 |
| Low registration x Intelligence | .00081 | .13 | .940 | .00081 |
| Sensation seeking x Intelligence | .00072 | .12 | .948 | .00072 |
| Sensory sensitivity x Intelligence | .0050 | .84 | .471 | .0050 |
| Sensation avoiding x Intelligence | .0044 | .73 | .534 | .0044 |
| Low registration quadratic term x Intelligence | .0067 | 1.1 | .344 | .0067 |
| Sensation seeking quadratic term x Intelligence | .0059 | .99 | .398 | .0059 |
| Sensory sensitivity quadratic term x Intelligence | .0060 | 1.0 | .390 | .0060 |
| Sensation avoiding quadratic term x Intelligence | .0094 | 1.6 | .193 | .0094 |
| Teacher-reported model (*n*=296) | | | | |
|  | *Pillai’s Trace* | *F(3,286)* | *p* | *ηp²* |
| Low registration | .0039 | .34 | .800 | .0039 |
| Sensation seeking | .011 | .96 | .413 | .011 |
| Sensory sensitivity | .014 | 1.2 | .316 | .014 |
| Sensation avoiding | .022 | 1.9 | .129 | .022 |
| Low registration quadratic term | .013 | 1.1 | .341 | .013 |
| Sensation seeking quadratic term | .0033 | .28 | .838 | .0033 |
| Sensory sensitivity quadratic term | .0074 | .64 | .592 | .0074 |
| Sensation avoiding quadratic term | .0044 | .38 | .767 | .0044 |
| Sex | .043 | 3.9 | .010† | .043 |
| Infant neurodevelopment | .0043 | .37 | .777 | .0043 |
| Intelligence | .046 | 4.2 | .007† | .046 |
| Low registration x Sex | .0033 | .29 | .834 | .0033 |
| Sensation seeking x Sex | .0081 | .70 | .552 | .0081 |
| Sensory sensitivity x Sex | .015 | 1.3 | .273 | .015 |
| Sensation avoiding x Sex | .010 | .85 | .470 | .010 |
| Low registration quadratic term x Sex | .022 | 1.9 | .130 | .022 |
| Sensation seeking quadratic term x Sex | .0030 | .26 | .856 | .0030 |
| Sensory sensitivity quadratic term x Sex | .014 | 1.2 | .297 | .014 |
| Sensation avoiding quadratic term x Sex | .0016 | .14 | .936 | .0016 |
| Low registration x Infant neurodevelopment | .015 | 1.30 | .276 | .015 |
| Sensation seeking x Infant neurodevelopment | .0070 | .61 | .611 | .0070 |
| Sensory sensitivity x Infant neurodevelopment | .0095 | .82 | .484 | .0095 |
| Sensation avoiding x Infant neurodevelopment | .0040 | .35 | .791 | .0040 |
| Low registration quadratic term x Infant neurodevelopment | .015 | 1.3 | .280 | .015 |
| Sensation seeking quadratic term x Infant neurodevelopment | .016 | 1.4 | .257 | .016 |
| Sensory sensitivity quadratic term x Infant neurodevelopment | .013 | 1.1 | .334 | .013 |
| Sensation avoiding quadratic term x Infant neurodevelopment | .016 | 1.4 | .245 | .016 |
| Low registration x Intelligence | .0064 | .56 | .644 | .0064 |
| Sensation seeking x Intelligence | .017 | 1.5 | .228 | .017 |
| Sensory sensitivity x Intelligence | .015 | 1.3 | .262 | .015 |
| Sensation avoiding x Intelligence | .0018 | .15 | .929 | .0018 |
| Low registration quadratic term x Intelligence | .013 | 1.1 | .332 | .013 |
| Sensation seeking quadratic term x Intelligence | .014 | 1.2 | .300 | .014 |
| Sensory sensitivity quadratic term x Intelligence | .0054 | .47 | .705 | .0054 |
| Sensation avoiding quadratic term x Intelligence | .0051 | .44 | .722 | .0051 |

*Note.* †P-value does not remain significant after correcting for multiple testing. The bold p-value indicates statistical significance.

Table S3. *Multivariate effects of the multivariate polynomial regression models examining the relation between sensory processing and neurodevelopmental traits, while adjusting for the child’s sex (boy/girl), infant neurodevelopment (typical/atypical) and intelligence level (continuous) at 4-5 years.*

| Parent-reported model (*n*=536) | | | | |
| --- | --- | --- | --- | --- |
|  | *Pillai’s Trace* | *F(3,524)* | *p* | *ηp²* |
| Low registration | .075 | 14 | **<.001** | .075 |
| Sensation seeking | .10 | 19 | **<.001** | .10 |
| Sensory sensitivity | .035 | 6.4 | **<.001** | .035 |
| Sensation avoiding | .017 | 3.0 | .031† | .017 |
| Low registration quadratic term | .042 | 7.7 | **<.001** | .042 |
| Sensation seeking quadratic term | .031 | 5.5 | **<.001** | .031 |
| Sensory sensitivity quadratic term | .0033 | .56 | .630 | .0033 |
| Sensation avoiding quadratic term | .012 | 2.1 | .094 | .012 |
| Sex | .021 | 3.8 | .011† | .021 |
| Infant neurodevelopment | .0022 | .39 | .762 | .0022 |
| Intelligence | .20 | 42 | **<.001** | .20 |
| Teacher-reported model (*n*=296) | | | | |
|  | *Pillai’s Trace* | *F(3,286)* | *p* | *ηp²* |
| Low registration | .046 | 4.5 | **.004** | .046 |
| Sensation seeking | .073 | 7.4 | **<.001** | .073 |
| Sensory sensitivity | .012 | 1.1 | .344 | .012 |
| Sensation avoiding | .031 | 3.0 | .031† | .031 |
| Low registration quadratic term | .012 | 1.2 | .316 | .012 |
| Sensation seeking quadratic term | .039 | 3.8 | .011† | .039 |
| Sensory sensitivity quadratic term | .019 | 1.9 | .135 | .019 |
| Sensation avoiding quadratic term | .0041 | .39 | .764 | .0041 |
| Sex | .061 | 6.1 | **<.001** | .061 |
| Infant neurodevelopment | .0055 | .52 | .671 | .0055 |
| Intelligence | .14 | 15 | **<.001** | .14 |

*Note.* †P-value does not remain significant after correcting for multiple testing. Bold p-values indicate statistical significance.

Supplementary Table S4. *The association between sensory processing^1^ and neurodevelopmental disorder traits adjusted for sex, neurodevelopment in infancy and intelligence*

|  | Model with parent-reported ASD/ADHD traits  (*n* = 536) | | | | Model with teacher-reported ASD/ADHD traits  (*n* = 296) | | | |
| --- | --- | --- | --- | --- | --- | --- | --- | --- |
| Low registration | Pillai’s Trace=.073, *F*(3,524)=13.8, ***p*<.001**, *ηp²*=.073 | | | | Pillai’s Trace=.036, *F*(3,286)=3.53, *p*=.015, *ηp²*=.036† | | | |
|  | *b* (*SE*) |  | 95%*CI* | *ηp²* | *b* (*SE*) |  | 95%*CI* | *ηp²* |
| ASD traits | -.57 (.17) |  | **[-.89, -.26]** | .024 | -.0058 (.028) |  | [-.050, .039] | .00023 |
| ADHD traits | -.37 (.075) |  | **[-.52, -.23]** | .047 | -.15 (.094) |  | [-.34, .038] | .0085 |
| DCD traits | -.13 (.032) |  | **[-.19, -.067]** | .030 | -.13 (.042) |  | [-.21, -.044] | .030 |
| Sensation seeking | Pillai’s Trace=.091, *F*(3,524)=17.4, ***p*<.001**, *ηp²*=.091 | | | | Pillai’s Trace=.047, *F*(3,286)=4.70, ***p*=.003**, *ηp²*=.047 | | | |
| ASD traits | -.19 (.065) |  | **[-.32, -.067]** | .017 | -.010 (.011) |  | [-.028, .0085] | .0038 |
| ADHD traits | -.19 (.028) |  | **[-.25, -.13]** | .073 | -.13 (.048) |  | **[-.21, -.054]** | .037 |
| DCD traits | .030 (.013) |  | **[.0041, .055]** | .010 | .019 (.017) |  | [-.015, .053] | .0043 |
| Sensory sensitivity | Pillai’s Trace=.042, *F*(3,524)=7.66, ***p*<.001**, *ηp²*=.042 | | | | Pillai’s Trace=.0028, *F*(3,286)=.265, *p*=.851, *ηp²*=.0028 | | | |
| ASD traits | -.36 (.12) |  | **[-.56, -.15]** | .022 | -.0068 (.020) |  | [-.040, .027] | .00055 |
| ADHD traits | -.19 (.048) |  | **[-.29, -.097]** | .029 | -.043 (.084) |  | [-.19, .10] | .0012 |
| DCD traits | -.044 (.022) |  | **[-.086, -.0023]** | .0081 | -.024 (.033) |  | [-.087, .039] | .0020 |
| Sensation avoiding | Pillai’s Trace=.022, *F*(3,524)=3.88, ***p=*.009**, *ηp²*=.022 | | | | Pillai’s Trace=.031, *F*(3,286)=3.01, *p*=.031, *ηp²*=.031† | | | |
| ASD traits | -.22 (.095) |  | **[-.38, -.068]** | .015 | -.012 (.013) |  | [-.037, .013] | .0033 |
| ADHD traits | .020 (.039) |  | [-.051, .092] | .0060 | .11 (.059) |  | [.0050, .22] | .015 |
| DCD traits | .016 (.015) |  | [-.015, .047] | .0020 | .023 (.021) |  | [-.023, .070] | .0033 |
| Low registration quadratic term | Pillai’s Trace=.054, *F*(3,524)=10.0, ***p*<.001**, *ηp²*=.054 | | | | †† | | | |
| ASD traits | .012 (.010) |  | [-.00053, .025] | .0067 |  |  |  |  |
| ADHD traits | -.010 (.0032) |  | **[-.016, -.0042]** | .021 |  |  |  |  |
| DCD traits | -.0044 (.0015) |  | **[-.0070, -.0017]** | .020 |  |  |  |  |
| Sensation seeking quadratic term | Pillai’s Trace=.042, *F*(3,524)=7.64, ***p<*.001**, *ηp²*=.042 | | | | †† | | | |
| ASD traits | .0063 (.0038) |  | **[.0022, .010]** | .017 |  |  |  |  |
| ADHD traits | .0042 (.0010) |  | **[.0023, .0061]** | .034 |  |  |  |  |
| DCD traits | .00074 (.00043) |  | [-.000091, .0016] | .0058 |  |  |  |  |
| Sex | Pillai’s Trace=.021, *F*(3,524)=3.72, ***p*=.011**, *ηp²*=.021 | | | | Pillai’s Trace=.059, *F*(3,286)=5.99, ***p*<.001**, *ηp²*=.059 | | | |
| ASD traits | -2.4 (1.1) |  | **[-4.6, -.22]** | .0088 | -.64 (.18) |  | [-.98, -.29] | .043 |
| ADHD traits | -.80 (.51) |  | [-1.8, .22] | .0045 | -2.4 (.75) |  | [-3.9, -.88] | .033 |
| DCD traits | -.60 (.23) |  | **[-1.0, -.16]** | .013 | -.70 (.34) |  | [-1.4, -.053] | .015 |
| Infant neurodevelopment | Pillai’s Trace=.0031, *F*(3,524)=.545, *p*=.652, *ηp²*=.0031 | | | | Pillai’s Trace=.0062, *F*(3,286)=.595, *p*=.619, *ηp²*=.0062 | | | |
| ASD traits | .80 (1.1) |  | [-1.4, 3.0] | .0095 | -.20 (.18) |  | [-.55, .16] | .0042 |
| ADHD traits | .54 (.52) |  | [-.48, 1.6] | .0021 | .18 (.77) |  | [-1.3, 1.7] | .00019 |
| DCD traits | -.11 (.23) |  | [-.55, .34] | .00042 | -.027 (.34) |  | [-.69, .64] | .000022 |
| Intelligence | Pillai’s Trace=.194, *F*(3,524)=42.1, ***p*<.001**, *ηp²*=.194 | | | | Pillai’s Trace=.134, *F*(3,286)=14.8, ***p*<.001**, *ηp²*=.134 | | | |
| ASD traits | -.26 (.041) |  | **[-.34, -.18]** | .072 | -.0056 (.0079) |  | [-.018, .0070] | .0027 |
| ADHD traits | -.044 (.018) |  | **[-.081, -.0073]** | .010 | -.081 (.027) |  | [-.13, -.027] | .029 |
| DCD traits | -.080 (.0083) |  | **[-.096, -.064]** | .155 | -.076 (.012) |  | [-.10, -.052] | .122 |

*Note.* ^1^ Low registration, sensation seeking, sensory sensitivity and sensation avoiding. † P-value does not remain significant after correcting for multiple testing. †† variable not included in the model, as the prior model showed that the multivariate effect of this variable was not significant. ASD = Autism Spectrum Disorder, ADHD = Attention Deficit Hyperactivity Disorder, DCD = Developmental Coordination Disorder. Lower scores on sensory quadrants indicate more sensory-related behaviour. For all neurodevelopmental disorder traits, higher values indicate more severe difficulties. Bold values indicate statistical significance.

Supplementary Table S5. *Comparison mean scores sensory quadrants between the current sample and the Sensory Profile Dutch norm sample.*

|  | Current sample (*n* = 542)  *M (SD)* | Dutch norm sample (*n* = 667)  *M* (*SD)* | *p* |
| --- | --- | --- | --- |
| Low registration | 67.66 (7.20) | 67.58 (5.50) | .831 |
| Sensation seeking | 109.29 (14.26) | 107.98 (12.35) | .092 |
| Sensory sensitivity | 87.88 (9.96) | 88.07 (7.64) | .715 |
| Sensation avoiding | 124.63 (13.14) | 122.32 (11.74) | **.001** |

*Note.* Bold p-value indicates statistical significance.
